# 

**Published:** 1896-05

**Authors:** 


					﻿CONTENTS
ORIGINAL ARTICLES—
The Treatment of Skin Disfigurements by Electrolysis.
By M. B. Hutchins, M. D...................   .'...... 211
Pertaining to Circumcision. By I. N. Love, M. D., St. Louis. 215
SOCIETY REPORTS—
The Medical Association of Georgia..................... 220
SELECTIONS AND ABSTRACTS—
Specialist vs. General Practitioner.................... 233
Observations in European Surgery....................... 238
Penetrating Wounds of the Chest........................ 240
Transplantation of Teeth............................... 240
The Passing of the Ovary............................... 242
EDITORIAL—
State Medical Association Meeting at	Augusta......... 243
Wm. C. Cooper, of Cleves, Ohio,	Poet............ 243
The X Ray......................................  246
NOTES—..............................................  251
BOOK REVIEWS......................................... 253
SPECIAL NOTES—..................................... ‘	254
Prescriptions.................................... 260
				

